# Hypoxia Inducible Factor-1α in Osteochondral Tissue Engineering

**DOI:** 10.1089/ten.teb.2019.0283

**Published:** 2020-04-16

**Authors:** Dheraj K. Taheem, Gavin Jell, Eileen Gentleman

**Affiliations:** ^1^ Centre for Craniofacial and Regenerative Biology, King's College London, London, United Kingdom.; ^2^ Division of Surgery and Interventional Sciences, University College London, London, United Kingdom.

**Keywords:** HIF-1α, hypoxia, cartilage, osteochondral tissue engineering

## Abstract

Damage to osteochondral (OC) tissues can lead to pain, loss of motility, and progress to osteoarthritis. Tissue engineering approaches offer the possibility of replacing damaged tissues and restoring joint function; however, replicating the spatial and functional heterogeneity of native OC tissue remains a pressing challenge. Chondrocytes in healthy cartilage exist in relatively low-oxygen conditions, while osteoblasts in the underlying bone experience higher oxygen pressures. Such oxygen gradients also exist in the limb bud, where they influence OC tissue development. The cellular response to these spatial variations in oxygen pressure, which is mediated by the hypoxia inducible factor (HIF) pathway, plays a central role in regulating osteo- and chondrogenesis by directing progenitor cell differentiation and promoting and maintaining appropriate extracellular matrix production. Understanding the role of the HIF pathway in OC tissue development may enable new approaches to engineer OC tissue. In this review, we discuss strategies to spatially and temporarily regulate the HIF pathway in progenitor cells to create functional OC tissue for regenerative therapies.

**Impact statement:**

Strategies to engineer osteochondral (OC) tissue are limited by the complex and varying microenvironmental conditions in native bone and cartilage. Indeed, native cartilage experiences low-oxygen conditions, while the underlying bone is relatively normoxic. The cellular response to these low-oxygen conditions, which is mediated through the hypoxia inducible factor (HIF) pathway, is known to promote and maintain the chondrocyte phenotype. By using tissue engineering scaffolds to spatially and temporally harness the HIF pathway, it may be possible to improve OC tissue engineering strategies for the regeneration of damaged cartilage and its underlying subchondral bone.

## Introduction

Cartilage has a poor capacity for self-repair after injury, which can lead to joint pain, immobility, and eventually osteoarthritis (OA). By repairing cartilage, it may be possible to restore joint function and prevent the development of OA. Tissue engineering (TE), a field whose primary aim is to form new tissue,^1^ has the potential to revolutionize treatments for cartilage damage. Native cartilage's primary function is to cushion bones and support the smooth movement of articular joints. Cartilage achieves this by seamlessly integrating with its underlying bone. Therefore, cartilage TE strategies often aim to engineer bone and cartilage together to create osteochondral (OC) constructs that can integrate with the supportive subchondral bone. However, engineering such disparate tissues in a single TE construct is challenging as cartilage is dominated by a collagen type II/proteoglycan-rich matrix with embedded chondrocytes, while the underlying bone comprises a mineralized collagen type I structure that is maintained by osteocytes. However, fundamental to both the development and maintenance of native OC tissue is oxygen pressure. Oxygen pressure is low in native cartilage, but higher in subchondral bone. The cellular response to oxygen pressure, which is mediated by the hypoxia inducible factor (HIF) pathway, is central in controlling the differentiation of progenitor cells during development, their production of appropriate extracellular matrix (ECM), and maintenance of their correct phenotype. Therefore, by creating TE scaffolds that can spatially harness the cellular response to oxygen pressure, it may be possible to effectively engineer functional OC tissue. In this study, we review the role of the HIF pathway in OC development and maintenance and discuss its potential for use in OC TE.

## The Effect of Oxygen Pressure and the HIF Pathway in OC Tissue Development and Maintenance

**FIG. 1.**
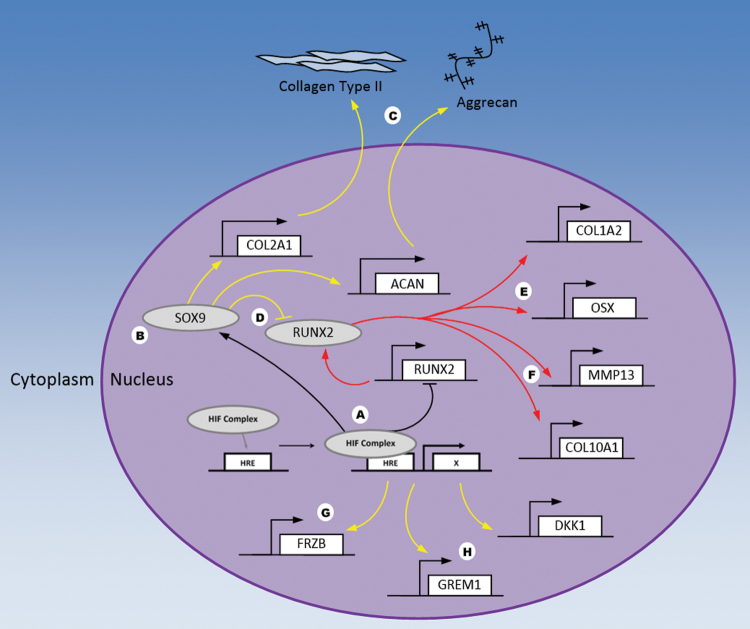
HIF pathway-mediated regulation of OC progenitor cells. Under hypoxic conditions, the HIF complex binds to its response elements (HREs) on target genes **(A)** where it acts to enhance *SOX9* expression and activity **(B)**, resulting in increased production of cartilage ECM **(C)**, dampened activity of *RUNX2* **(D)**, and reduced expression of genes involved in osteogenesis **(E)** and chondrocyte hypertrophy **(F)**. HIF has also been shown to increase expression of glycolytic enzymes **(G)** as well as Wnt antagonists involved in delaying chondrocyte hypertrophy **(H)**. *Yellow arrows* indicate pathways/genes that are activated, while *red arrows* show pathways/genes that are inhibited under hypoxic conditions. ECM, extracellular matrix; HIF, hypoxia inducible factor; HRE, hypoxia-responsive element; OC, osteochondral. Color images are available online.

The cellular response to oxygen plays an important role in both the development and maintenance of OC tissue^2–7^ and is primarily mediated through the HIF pathway (Fig. 1). Under normoxic conditions, HIF-1α, the oxygen-responsive subunit of the HIF complex, is continually degraded. However, under hypoxic conditions, HIF-1α accumulates within the cytoplasm and translocates to the nucleus where it regulates expression of target (hypoxia-responsive element, HRE) genes. Moreover, hypoxia enhances the binding between HIF-1α and its transcriptional cofactors, which further augments HIF complex-mediated regulation of gene expression.^8^ During development, the HIF pathway plays fundamental roles in directing the differentiation of OC progenitors. In general, increased HIF-1α stabilization (under low-oxygen conditions) stimulates a prochondrogenic, antiosteogenic, and antihypertrophic transcriptome.^9^ This effect is reversed in the presence of higher oxygen concentrations as HIF-1α is degraded, which promotes a more hypertrophic/osteoblastic fate.

### The Role of HIF-1α in Differentiation of OC Progenitor Cells, Their Survival, and Phenotypic Maintenance

During development, articular cartilage forms during endochondral ossification,^10^ the process by which the axial skeleton is created. To achieve this, a condensed population of mesenchymal precursors form the initial cartilaginous anlage, which is subsequently infiltrated by the vasculature and ossified.^11^ Concomitant with this process, cartilage is maintained at the ends of long bones and within it, a population of chondrocytes, which will go on to form the articular cartilage. HIF-1α is essential in this process as under the low-oxygen conditions of the developing growth plate, knockdown of HIF-1α results in chondrocyte cell death.^2^ Moreover, knockdown of the enzyme required for HIF-1α degradation in the growth plate deregulates mesenchymal precursor and chondrocyte proliferation^12,13^ and its conditional inactivation in the developing mouse limb bud mesenchyme negatively impacts both the formation of cartilage and joint development.^7^ When HIF-1α is conditionally inactivated, expression of *SOX9*, the master transcriptional regulator of chondrogenesis, and its downstream targets, the genes that encode collagen type II and aggrecan, the main constituents of cartilage tissue, are all reduced.^7,9^ *In vitro* activation of the HIF pathway has similarly been shown to upregulate *SOX9* expression and that of its downstream targets in murine^14^ and rat^15^ mesenchymal stromal cells (MSCs) as well as in human articular chondrocytes (hACs).^16^ Furthermore, engineering murine MSCs to stably express HIF-1α under normoxic conditions has been shown to potentiate their BMP2-induced chondrogenic differentiation.^17^

In addition to driving the differentiation of progenitors, hypoxia and HIF-1α also play a role in maintaining cells' chondrogenic phenotypes by preventing their hypertrophic or osteogenic differentiation. During endochondral ossification, signaling gradients, including those triggered by oxygen pressure, are responsible for retaining populations of chondrocytes in their nonhypertrophic state, priming them for a permanent, articular chondrocyte fate.^18^ Hypoxia and HIF-1α achieve this in hACs by downregulating the expression of hypertrophic fibroblast-like markers such as *COL1A1* and *COL3A1*.^19^ Moreover, hypoxia suppresses the expression of matrix metalloproteinases (MMPs) and aggrecanases in hACs, both of which degrade the cartilage matrix.^20^ Similarly, human and other mammalian cartilage explants cultured under hypoxic conditions show HIF-1α-mediated suppression of cartilage catabolism by a disintegrin and metalloproteinase with thrombospondin motifs (ADAMTS-5) and MMP-13.^21^ The ability of HIF signaling to promote a stable articular phenotype is also supported by observations that hypoxia enhances the expression of antihypertrophic Wnt antagonists,^22^ and HIF-1α conditional knockout in developing cartilage results in reduced expression of Wnt9a and GDF5.^9^

### The Role of HIF-1α in Cartilage ECM Formation

**FIG. 2.**
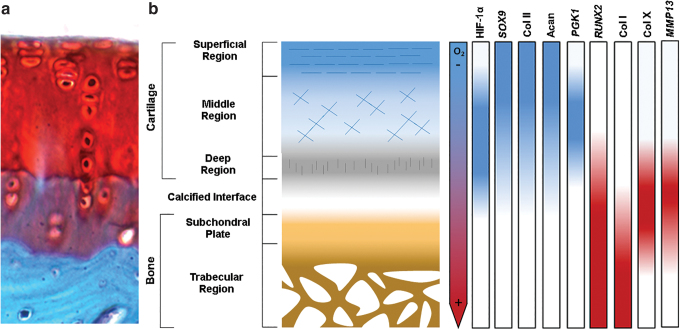
Oxygen, HIF-1α, transcription factor, and ECM gradients in OC tissue. Oxygen pressure is low in native cartilage, but high in the subchondral bone. The cellular response to these varying oxygen pressures, which are mediated by HIF-1α, plays a role in regulating transcription factors important in progenitor cell differentiation toward chondrogenic and osteogenic phenotypes and in promoting the production of proteins that are abundant in cartilage compared with bone. **(a)** A histological section of the OC tissue from a rabbit metacarpophalangeal joint stained with hematoxylin, Safranin O, and fast green. Cartilage appears *red*, calcified cartilage appears *dark green*, and bone appears *blue*. Image is adapted from^106^ (CC-BY 2.0). **(b)** A schematic of OC tissue showing expression gradients of HIF-1α, transcription factors, proteins, and enzymes. Factors in *blue* are upregulated or promoted in response to low oxygen pressures, while those in *red* are downregulated.^2,107–112^ col II, collagen type II; acan, aggrecan; col I, collagen type I; col X, collagen type X. Color images are available online.

Not only do physiological hypoxia and the HIF pathway play important roles in regulating the differentiation of OC progenitor cells but they also appear to drive the formation of appropriate ECM. The ECM of native cartilage is dominated by a combination of collagen type II and proteoglycans, and hypoxia and the HIF pathway have been shown to regulate the formation of this matrix (Fig. 2). For example, physiological hypoxia enhances the production of cartilage-specific ECM in cultured hACs when compared with that formed under normoxic conditions.^23^ Similarly, hACs in pellet cultures synthesize collagen fibrils with more ordered morphologies when cultured in 5% oxygen compared with under normoxic conditions.^16,21,23^ Similar observations have been made in chondrocytes embedded in alginate hydrogels^24^ and seeded on 3D PLGA scaffolds.^25^ Moreover, human bone marrow-derived MSCs and hACs pretreated with hypoxia before encapsulation in alginate and implantation in a nude mouse model showed enhanced cartilage ECM formation compared with that observed when cells were precultured under normoxic conditions.^26^

Regulation of ECM synthesis is dependent on the activity of HIF-1α as its conditional knockdown in developing murine limbs results in abnormal ECM morphology as well as reduced production of proteoglycans and collagen type II in the growth plate.^7,9,27^ Similarly, stabilizing HIF-1α chemically by inhibiting its ubiquitination and degradation causes hACs to increase their secretion of collagen type II and aggrecan.^28^ Hypoxia has also been shown to increase cartilage-like ECM production in MSCs.^29^ Indeed, delivery of a HIF-1α expression vector enhances the expression of genes for both collagen type II and the proteoglycan aggrecan, as well as a panel of chondrogenic transcription factors.^26^ Moreover, stabilization of HIF-1α promotes the formation of cartilage-specific ECM by both upregulating the expression of *SOX9* and through post-translational modifications to collagen type II.^30^ HIF-1α's role in regulating ECM production is thought to be mediated by its regulation of collagen prolyl 4-hydroxylase, which is required for the addition of 4-hydroxyproline residues to collagen fibrils, allowing them to form triple helices.^30^ In addition, the HIF pathway is also thought to control expression of lysyl oxidase, an enzyme required for the cross-linking of collagen triple helices.^31,32^

## Strategies for OC TE

A common approach to engineer cartilage tissue is to mimic aspects of the native, *in vivo* cellular microenvironment in 3D scaffolds seeded with appropriate progenitor cells.^33–35^ A clinical example of this is matrix-assisted, autologous chondrocyte transplantation/implantation (MACT/MACI), which involves seeding autologous chondrocytes in a 3D scaffold before surgical implantation. However, despite providing chondrocytes with a 3D environment,^36^ the MACT matrix often does not adequately mimic many microenvironmental conditions within native cartilage, including local oxygen pressure. Moreover, integration with the underlying subchondral bone remains an issue.

Because of these drawbacks, researchers have attempted to engineer OC tissue that can seamlessly integrate with the underlying subchondral bone. This can be accomplished either by engineering a monophasic cartilage construct and then relying on the native local environment to drive ossification at the bone interface or by engineering a bone–cartilage construct that contains both tissues before implantation. Monophasic cartilage TE approaches have been applied widely. For example, Koga *et al.* created cartilage using synovium-derived MSCs. When implanted in a rabbit model, MSCs produced extensive cartilage matrix, while cells adjacent to the subchondral bone differentiated into osteoblasts.^37^ However, such endogenous processes are uncontrolled, and movement of the tidemark, the interface between the bone and the cartilage, has been observed. Therefore, others have attempted to create OC constructs that contain either bone and cartilage or bone, cartilage, and an interfacial region. Engineering a single construct that contains bone and cartilage, however, remains challenging because of the tissues' distinct compositions, architectures, and cellular microenvironments. One strategy to address this is to create biphasic scaffolds formed from separate osteogenic and chondrogenic constructs that are later combined. Such strategies have been tested *in vitro*^38^ and in large animal models.^39^ However, unlike in native OC tissue in which a calcified hypertrophic zone exists between the bone and cartilage, in many biphasic scaffolds, an abrupt artificial interface may form, which may impact the construct's mechanical integrity. For example, when Grayson *et al.* synthesized a biphasic construct by placing agarose within a decellularized bone scaffold, they reported the absence of a hypertrophic transition zone.^40^

Researchers have also attempted to form triphasic scaffolds, which contain an interfacial region between the cartilage and bone scaffolds, to more faithfully recapitulate the native OC interface. For example, Da *et al.* formed a compact interfacial layer by placing poly(lactic-co-glycolic acid)-β-tricalcium phosphate between the chondrogenic and bony components of a biphasic scaffold. They observed enhanced mechanical properties in the interface-containing scaffolds compared with those that lacked the interfacial region, as well as increased OC tissue regeneration in a rabbit model.^41^ Similarly, Kon *et al.* formed triphasic scaffolds by varying the ratios of type I collagen and hydroxyapatite in their constructs. When tested in 15 patients with cartilage lesions, they were able to demonstrate safety and short-term follow-up appeared promising.^42^ Nevertheless, like biphasic scaffolds, triphasic scaffolds may still not fully recapitulate the native tissue's seamless interface and thus may separate *in vivo*. This has been observed in polycaprolactone/alginate scaffolds upon subcutaneous implantation in a rat model, where the osteogenic and chondrogenic portions often became separated.^43^ Moreover, biphasic and triphasic scaffolds may require separate chondrogenic and osteogenic culture conditions, which may create logistical challenges for their scale-up and clinical use.

Alternatives to multiphasic scaffolds are continuous OC scaffolds designed to enable synchronous formation of both cartilage and bone with a seamless transition, mimicking the calcified hypertrophic interface in native OC tissue. Continuous scaffolds may also preclude the need for separate culture conditions as they can be designed to differentiate a single progenitor population down different lineages depending on location within the biomaterial construct. For example, Harley *et al.* created continuous OC scaffolds by lyophilizing mineralized and unmineralized type I collagen–glycosaminoglycan suspensions to form a natural interface.^44^ Researchers have also achieved continuous scaffolds by creating morphogen gradients. For example, Wang *et al.* utilized BMP2 and IGF-1-containing microspheres to create inverse gradients in alginate hydrogels and observed corresponding differentiation of encapsulated human MSCs (hMSCs) down chondrogenic and osteogenic lineages.^45^ Similarly, Mohan *et al.* utilized inverse gradients of microspheres containing BMP2 and TGF-β1. When implanted in a rat femoral defect model, they showed region-specific regeneration of cartilage and bone and formation of a stable interface.^46^

## Exploiting the HIF-1α Regulatory Network for OC TE

As oxygen gradients form during OC tissue development and aid in the maintenance of OC tissue in the adult, controlling oxygen pressure may be an effective strategy to engineer OC tissue. Researchers have described strategies to locally regulate oxygen pressure *in situ* within biomaterials. For example, oxygen-releasing molecules such as perfluorocarbons^47^ and hemoglobin^48^ or myglobin^49^ can be incorporated into biomaterials to increase local oxygen levels, using strategies amenable for OC TE. Similarly, manganese dioxide nanoparticles^50^ and calcium peroxide^51^ can be used to generate oxygen within a TE construct. Alternatively, oxygen scavengers can mediate the opposite effect and lower local levels of oxygen. Indeed, it is possible to locally decrease the oxygen pressure within a biomaterial either by incorporating various molecules^52^ or simply by limiting oxygen diffusion, which has been shown to stimulate the chondrogenesis of progenitor cells.^53^

**FIG. 3.**
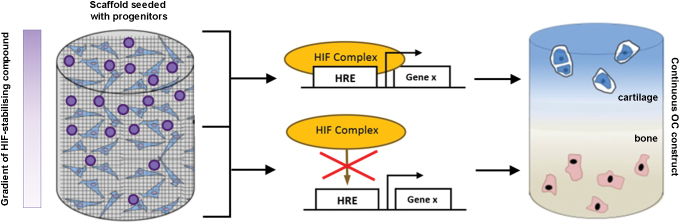
Exploiting the HIF pathway in OC TE. An example of a progenitor-seeded biomaterial scaffold containing a gradient of an HIF-1α-stabilizing compound. During differentiation and tissue formation, the differential levels of the HIF-1α-stabilizing agent promote a continuous interface that mimics that in native OC tissue. The resulting continuous OC construct would then contain spatially restricted regions of articular cartilage and subchondral bone. TE, tissue engineering. Color images are available online.

However, regulating oxygen itself may not be ideal as hypoxia is also known to cause oxidative stress, prompt potentially undesirable effects on cell metabolism, and negatively impact cell growth and viability,^54^ all of which may be detrimental to forming tissue. An alternative approach is to stabilize HIF-1α under normoxic conditions as this has the potential to provide the beneficial prochondrogenic effects of hypoxia, but in a more controlled and potentially less deleterious manner. Indeed, as stabilization of HIF-1α enhances the chondrogenic differentiation of progenitor cells,^55^ minimizes chondrocyte hypertrophy, and stimulates the production of cartilage-like ECM, manipulating its regulatory network within TE scaffolds may be an effective strategy to engineer OC tissue. A number of compounds have been reported to ectopically stabilize HIF-1α at normoxia and thus stimulate cellular responses that mimic those elicited by low oxygen pressure. Therefore, by incorporating these HIF mimetics into the chondrogenic region of a TE scaffold, it may be possible to stimulate progenitor cells to undergo region-specific formation of articular cartilage (Fig. 3). To accomplish this, appropriate components of the HIF complex that can be targeted pharmacologically need to be recognized and compounds that act against them identified and incorporated into scaffolds.

### Regulation of the HIF Transcriptional Complex

The α subunit of HIF exists in two forms—HIF-1α and HIF-2α—both of which play roles in the regulation of cartilage formation. Knockout of HIF-1α results in cell death and cartilage catabolism in the developing limb bud mesenchyme,^2,56^ and suppression of HIF-1α negatively impacts the production of cartilage-associated matrix proteins in cultured chondrocytes. HIF-2α, on the other hand, regulates endochondral ossification by mediating angiogenesis and ossification of the cartilage template^56^ and plays important roles in cartilage degradation during OA.^57^ Therefore, because of HIF-1α's central role in maintaining the chondrocyte phenotype and cartilage ECM,^30^ it is the more obvious target for OC TE strategies.

**FIG. 4.**
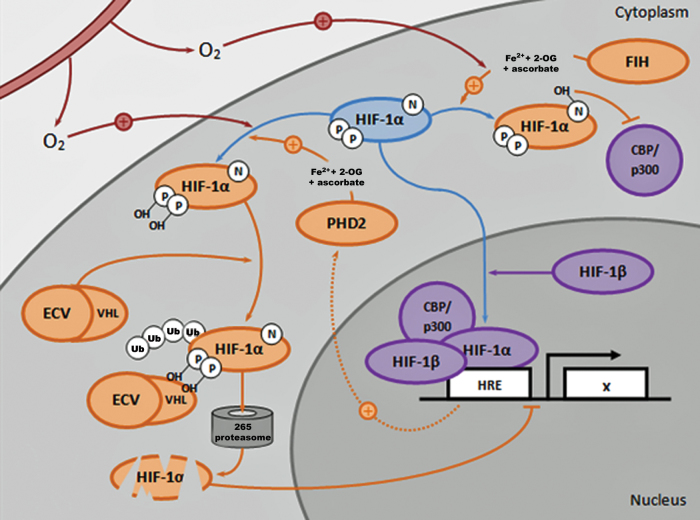
The PHD2-VHL signaling cascade: an opportunity to regulate the HIF pathway. In response to molecular oxygen (O_2_), FIH and PHD2 (in the presence of Fe^2+^, 2-OG, and ascorbate) hydroxylate specific amino acid residues on HIF-1α. FIH-mediated hydroxylation blocks cofactors CBP/p300 from binding to HIF-1α, thereby reducing HIF transcriptional activity. PHD2-mediated hydroxylation results in ubiquitination of the ODDD domain of HIF-1α by the VHL component of the ECV (elongin/culin/VHL) ubiquitin ligase complex, thus promoting degradation of HIF-1α in the 265 proteasome and reducing HIF transcriptional activity. Under hypoxic conditions, PHD2 and FIH activity are reduced, thus enabling HIF-1α to translocate and accumulate in the nucleus, where it activates expression of HIF target genes in the HRE as part of a transcriptional complex with HIF-1β, CBP/p300, and other cofactors. A negative feedback mechanism exists in which PHD2 expression is also enhanced by HIF activity. CBP, CREB-binding protein; FIH, factor inhibiting HIF; 2-OG, 2-oxoglutarate; ODDD, oxygen-dependent degradation domain; PHD2-VHL, prolyl hydroxylase 2–von Hippel-Lindau. Color images are available online.

Central to regulation of HIF-1α is the prolyl hydroxylase 2–von Hippel-Lindau (PHD2-VHL) signaling cascade^58^ (Fig. 4). At normoxia, PHD2 utilizes molecular oxygen and other cofactors to hydroxylate residues on the oxygen-dependent degradation domain (ODDD) of HIF-1α.^59^ The hydroxylated residues then serve as recognition motifs by the VHL tumor suppressor protein. As part of the E3 ubiquitin ligase complex, VHL binds and ubiquitinates the hydroxylated residues of HIF-1α, targeting the molecule for degradation by the proteasome.^60–62^ However, under hypoxic conditions, PHD2's ability to hydroxylate HIF-1α is diminished, enabling its cytosolic accumulation and nuclear translocation, where together with transcriptional cofactors, it activates expression of its target genes in the HIF complex.^63^ Other pathways central to regulating HIF-1α degradation are RACK1 and HSP90. HSP90 normally binds to HIF-1α, thus preventing its degradation. However, RACK1 can compete with HSP90 in its binding to HIF-1α and, in doing so, recruits the same ubiquitinating complex utilized by VHL, thereby resulting in PHD2/VHL-independent HIF-1α degradation.^64–66^

In addition to the PHD2-VHL pathway, HIF-1α also requires cofactors to be recruited to the HIF transcriptional complex to activate gene expression when bound to the HRE in target gene promoters. Two important factors in this complex are p300 and the CREB-binding protein (CBP).^67^ One key residue on HIF-1α involved in its binding with p300/CBP is asparagine-803 (Asn-803).^8^ Indeed, Asn-803 is also the target of another 2-oxoglutarate (2-OG)-utilizing hydroxylase, factor inhibiting HIF (FIH), which similarly regulates HIF transcriptional activity.^8^ FIH hydroxylates Asn-803 on HIF-1α, preventing the binding of p300/CBP to HIF-1α, and therefore disrupts the formation of a functional HIF transcriptional complex.^8^

### Harnessing the HIF Pathway for OC TE by Stabilizing HIF-1α

**FIG. 5.**
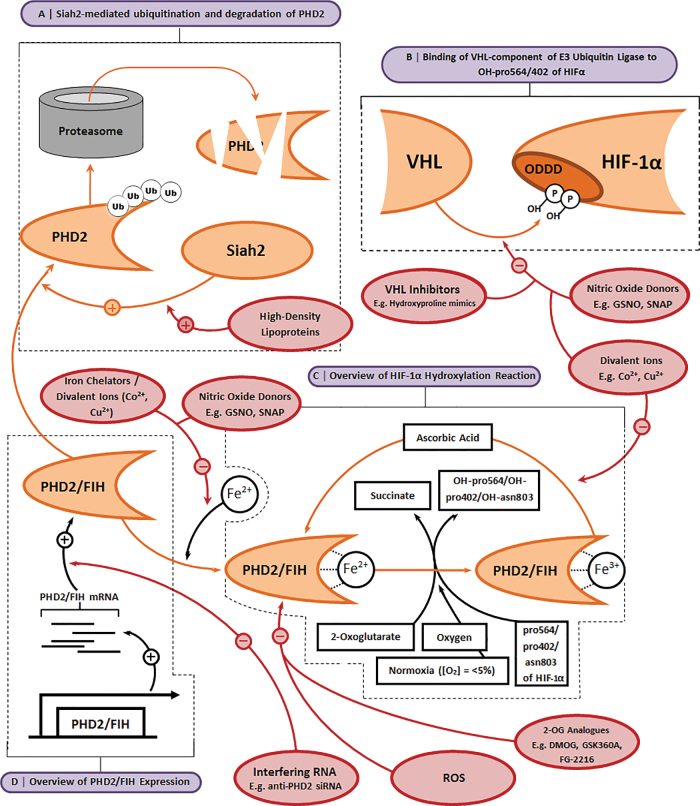
Pharmacological strategies to manipulate the HIF pathway. **(A)** High-density lipoproteins regulate HIF-1α levels through activation of Siah2, an E3 ubiquitin ligase, which targets PHD2 for proteasomal degradation. **(B)** Blocking binding of the VHL component of the E3 ubiquitin ligase to HIF-1α, following its proline hydroxylation, reduces ubiquitination and degradation. This can be achieved by molecules that bind and occupy the HIF-1α binding site of VHL or through the use of nitric oxide donors, which chemically modify VHL or the ODDD of HIF-1α. **(C)** HIF-1α hydroxylation reactions can be inhibited to reduce the subsequent ubiquitination and degradation of HIF-1α. For example, hydroxylation can be inhibited by specific hydroxylase inhibitors such as cofactor analogs, agents that sequester the Fe^2+^ that is required at the hydroxylase active sites, or ROS, which downregulate expression of PHD2. **(D)** HIF hydroxylases are susceptible to downregulation at the transcriptional level by interfering RNA molecules, which reduce their translation and thus HIF-1α hydroxylation. Asn803, asparagine-803; pro564/pro402, proline564/proline402; ROS, reactive oxygen species. Color images are available online.

Over the past two decades, there has been tremendous interest in identifying compounds that are able to stabilize HIF-1α and enhance its binding by transcriptional cofactors at normoxia (Fig. 5) for potential use as therapeutic agents to treat a range of conditions. The most common HIF mimetics include dimethyloxalylglycine (DMOG), desferrioxamine (DFX), and cobalt chloride (CoCl_2_), all of which target PHD2 and/or FIH.^68,69^ By targeting PHD2 and FIH, HIF mimetics reduce HIF-1α's prolyl and asparagine hydroxylation, reducing its subsequent degradation, and enhance its binding by transcriptional cofactors. DMOG acts through competition with 2-OG by engaging the binding pocket of the prolyl hydroxylase active site on both FIH and PHD2.^70^ DFX is an iron chelator and sequesters available Fe^2+^, which is required by both FIH and PHD2, thereby reducing their activity.^71^ CoCl_2_, on the other hand, may directly compete with Fe^2+^ binding to the PHD2 active site.^72^

Recent work to compare how DFX, DMOG, and CoCl_2_ impact hMSC chondrogenesis *in vitro* showed that DMOG upregulated expression of HIF target genes and induced a more chondrogenic transcriptional profile compared with either DFX or CoCl_2_.^55^ These observations suggest that hMSC chondrogenesis may be regulated by mechanisms with a greater dependence on 2-OG than Fe^2+^ availability and suggest that compounds that target 2-OG may be more effective for OC TE. These findings are supported by observations that cobalt, when released from a bioactive glass, reduces hMSC chondrogenic differentiation despite stabilizing HIF-1α.^73^ Sathy *et al.* have since exploited the hypoxia-mimicking properties of DMOG for cartilage TE by placing it within porcine-MSC-laden alginate hydrogels. They showed that DMOG-containing constructs enhanced MSC chondrogenesis *in vitro* and cartilage-like tissue formation *in vivo.*^74^ However, the HIF mimetic type, specificity, concentration, and duration of exposure may also play roles in their efficacy in promoting chondrogenesis, as highlighted by conflicting results in the literature. Indeed, while cobalt has been shown to promote chondrogenesis,^75^ others have demonstrated that it inhibits chondrogenesis^73^ and that this may be dependent on cell source.^76^

Nevertheless, although promising, the three most widely tested HIF mimetics lack a high degree of specificity for PHD2 FIH. Indeed, DMOG may also target similarly structured enzymes that are essential for formation of the collagen triple helix.^77^ Similarly, chelating Fe^2+^ ions or displacing them in enzymatic reactions lends a poor degree of control as iron is central in a range of other vital biological processes, including the mitochondrial respiratory chain or PHDs involved in collagen synthesis.^78^ More recently, screens have identified additional 2-OG inhibitors,^79^ including Kreb's cycle metabolites^80,81^ and metal chelators,^82^ some of which have been tested in clinical trials.^83^ GSK360A, for example, has been shown to improve ventricular remodeling following myocardial infarction,^84^ and FibroGen's FG-2216 alleviates erythropoietin deficiency in various anemic conditions.^85^ It will be particularly interesting to learn how these compounds influence cell behavior in OC TE applications.

Alternative approaches can also be used to ectopically stabilize HIF-1α by targeting the PHD2-VHL pathway. For example, high-density lipoproteins (HDLs) enhance HIF-dependent VEGF signaling through regulation of HIF-1α post-translational modification.^86,87^ HDLs act through activation of the ubiquitin ligase Siah2,^88^ which (when active) inhibits PHD2/PHD3, leading to HIF-1α accumulation.^87,89^ PHD2/FIH inhibitors designed to mimic cofactors that act with hydroxylases or interfering RNA molecules are promising tools in this regard as they can specifically target PHD2 and FIH. Indeed, RNAi against PHD2^28,90^ and native hypoxia-driven microRNA^91,92^ have been shown to enhance HIF-1α stabilization.

Nitric oxide (NO) has also been implicated as a regulator of HIF-1α,^93^ an effect that can also be induced by NO donors such as GSNO, SNAP, NAC, and DetaNONOATE, which similarly increase intracellular HIF-1α levels.^94^ These compounds modify the HIF-1α ODDD through N-nitrosylation and, in doing so, block VHL binding and subsequent HIF-1α ubiquitination.^95,96^ GSNO has also been shown to inhibit PHD2 and FIH activity, at least in part, by blocking the binding of Fe^2+^ to the active site, leading to similar levels of HIF-1α stabilization as those observed in CoCl_2_-treated cell cultures.^97,98^ SNAP similarly promotes HIF-dependent gene expression by inhibiting VHL-HIF-1α binding and FIH activity,^99^ and NAC has been shown to mimic the physiological effect of chronic hypoxia in murine, vascular, pulmonary endothelial cells through nitrosylation of proteins in the PHD2/VHL pathway.^100^ Nevertheless, like the HIF mimetics, targeting NO for OC TE is not specific as NO has a variety of biological roles.

Researchers have also attempted to target the HIF-1α-regulating effects of VHL.^101^ Peptides that mimic the hydroxylated ODDD of HIF-1α compete with native HIF-1α to bind to VHL, reducing HIF-1α ubiquitination.^102,103^ This is a particularly promising approach for OC TE as the peptide-based inhibitors can be highly specific. Calcium signaling is also a potential target. A calcium ionophore, which facilitates Ca^2+^ entry into the cell, has been shown to inhibit dimerization and activation of RACK1, thereby inhibiting its role in HIF-1α degradation.^104^ An alternative to reducing HIF-1α degradation is augmenting HIF-1α translation. Calcium ionophores or a calcium compound may tap into calcium's role in HIF-1α translation. However, the use of factors that enhance HIF-1α translation may be most effective when used in combination with those that inhibit HIF-1α degradation to increase overall levels of HIF-1α and increase biological function of the HIF transcriptional complex.

## Outlook

As hypoxia plays fundamental roles in development and maintenance of OC tissue, attempting to mimic its effects on progenitor cells may be an effective means to engineer OC tissue. Indeed, a biomaterial that is able to spatially control the intracellular stabilization and cofactor binding of HIF-1α may stimulate region-specific formation of articular cartilage where HIF-1α is active, while promoting the formation of a subchondral bone region where HIF-1α activity is depleted, all within a single construct cultured under normoxic conditions. Stabilization of HIF-1α at normoxia can be achieved by using HIF mimetics such as DMOG, DFX, and CoCl_2_. However, targeting the native regulatory signaling pathways that control intracellular levels of HIF-1α, such as the ODDD domain of HIF-1α, using peptides or RNAi may be an even more effective means to control intracellular levels of HIF-1α.

Utilizing HIF mimetics for OC TE will likely require them to be stably incorporated into scaffolds in a regional or gradient manner and for their controlled release. This could be achieved by tethering HIF mimetics directly to the scaffold or by incorporating soluble factors within degradable microspheres, whose locations within the scaffold are spatially controlled.^45^ Light-based chemistries could also be used to locally attach a HIF mimetic to a scaffold by applying differential levels of UV light along the length of a presynthesized biomaterial.^105^ Indeed, strategies to achieve localized delivery of HIF mimetics are already within reach and thus can be quickly incorporated into OC TE designs with the potential to deliver on the promise of OC TE to repair cartilage lesions and prevent OA.
